# 53 The Role of Body Mass Index in Hospitalized Burn Patients

**DOI:** 10.1093/jbcr/irad045.027

**Published:** 2023-05-15

**Authors:** Syed Saquib, Paul Chestovich, Carmen Flores, Joseph Carroll, Douglas Fraser, Jessica Tullington, Kavita Batra

**Affiliations:** Kirk Kerkorian School of Medicine at UNLV/UMC Lions Burn Care Center, Las Vegas, Nevada; Kirk Kerkorian School of Medicine at UNLV, Las Vegas, Nevada; Kirk Kerkorian School of Medicine at UNLV, Las Vegas, Nevada; Kirk Kerkorian School of Medicine at UNLV, Las Vegas, Nevada; Kirk Kerkorian School of Medicine at UNLV, Las Vegas, Nevada; Kirk Kerkorian School of Medicine at UNLV, Las Vegas, Nevada; Kirk Kerkorian School of Medicine at UNLV, Las Vegas, Nevada; Kirk Kerkorian School of Medicine at UNLV/UMC Lions Burn Care Center, Las Vegas, Nevada; Kirk Kerkorian School of Medicine at UNLV, Las Vegas, Nevada; Kirk Kerkorian School of Medicine at UNLV, Las Vegas, Nevada; Kirk Kerkorian School of Medicine at UNLV, Las Vegas, Nevada; Kirk Kerkorian School of Medicine at UNLV, Las Vegas, Nevada; Kirk Kerkorian School of Medicine at UNLV, Las Vegas, Nevada; Kirk Kerkorian School of Medicine at UNLV, Las Vegas, Nevada; Kirk Kerkorian School of Medicine at UNLV/UMC Lions Burn Care Center, Las Vegas, Nevada; Kirk Kerkorian School of Medicine at UNLV, Las Vegas, Nevada; Kirk Kerkorian School of Medicine at UNLV, Las Vegas, Nevada; Kirk Kerkorian School of Medicine at UNLV, Las Vegas, Nevada; Kirk Kerkorian School of Medicine at UNLV, Las Vegas, Nevada; Kirk Kerkorian School of Medicine at UNLV, Las Vegas, Nevada; Kirk Kerkorian School of Medicine at UNLV, Las Vegas, Nevada; Kirk Kerkorian School of Medicine at UNLV/UMC Lions Burn Care Center, Las Vegas, Nevada; Kirk Kerkorian School of Medicine at UNLV, Las Vegas, Nevada; Kirk Kerkorian School of Medicine at UNLV, Las Vegas, Nevada; Kirk Kerkorian School of Medicine at UNLV, Las Vegas, Nevada; Kirk Kerkorian School of Medicine at UNLV, Las Vegas, Nevada; Kirk Kerkorian School of Medicine at UNLV, Las Vegas, Nevada; Kirk Kerkorian School of Medicine at UNLV, Las Vegas, Nevada; Kirk Kerkorian School of Medicine at UNLV/UMC Lions Burn Care Center, Las Vegas, Nevada; Kirk Kerkorian School of Medicine at UNLV, Las Vegas, Nevada; Kirk Kerkorian School of Medicine at UNLV, Las Vegas, Nevada; Kirk Kerkorian School of Medicine at UNLV, Las Vegas, Nevada; Kirk Kerkorian School of Medicine at UNLV, Las Vegas, Nevada; Kirk Kerkorian School of Medicine at UNLV, Las Vegas, Nevada; Kirk Kerkorian School of Medicine at UNLV, Las Vegas, Nevada; Kirk Kerkorian School of Medicine at UNLV/UMC Lions Burn Care Center, Las Vegas, Nevada; Kirk Kerkorian School of Medicine at UNLV, Las Vegas, Nevada; Kirk Kerkorian School of Medicine at UNLV, Las Vegas, Nevada; Kirk Kerkorian School of Medicine at UNLV, Las Vegas, Nevada; Kirk Kerkorian School of Medicine at UNLV, Las Vegas, Nevada; Kirk Kerkorian School of Medicine at UNLV, Las Vegas, Nevada; Kirk Kerkorian School of Medicine at UNLV, Las Vegas, Nevada; Kirk Kerkorian School of Medicine at UNLV/UMC Lions Burn Care Center, Las Vegas, Nevada; Kirk Kerkorian School of Medicine at UNLV, Las Vegas, Nevada; Kirk Kerkorian School of Medicine at UNLV, Las Vegas, Nevada; Kirk Kerkorian School of Medicine at UNLV, Las Vegas, Nevada; Kirk Kerkorian School of Medicine at UNLV, Las Vegas, Nevada; Kirk Kerkorian School of Medicine at UNLV, Las Vegas, Nevada; Kirk Kerkorian School of Medicine at UNLV, Las Vegas, Nevada

## Abstract

**Introduction:**

Body mass index (BMI) has been identified as an independent risk factor for morbidity and mortality in hospitalized patients. The objective of this study was to investigate what role BMI played in hospitalized burn patients.

**Methods:**

Registry data for all burn patients over the age of 18 admitted to our ABA verified burn center between July 1, 2015 to December 31, 2020 was analyzed. Patients were categorized based on the World Health Organization’s definition of BMI. Age, gender, co-morbidities, race, ethnicity, total body surface area (TBSA) and mechanism of injury were included. Total length of stay, ICU length of stay, the need for a ventilator, and hospital disposition were also analyzed.

Categorical variables were compared using chi-square, and continuous using one-way ANOVA with post-hoc analysis. A multivariate logistic regression model was fit to generate adjusted odds ratios for the likelihood in-patient mortality with a p-value of < 0.05 was considered significant

**Results:**

A total of 1106 patients were included for analysis. Mean age was 48.8 years. Mean BMI was 27.7. 785 (71%) were male. The most common burn mechanism was flame (551, 49.8 %). The following was the distribution based on BMI: underweight 50 (4.5%), normal weight 381 (34.4%), overweight 337 (30.5%), Class 1 obesity 198 (17.9%), Class 2 obesity 81 (7.3%), and Class 3 obesity 59 (5.3%).

All classes of obese patients were more likely to have hypertension (P < 0.001). Class 2 and 3 obese patients were more likely to have diabetes mellitus (P < 0.001). Underweight patients were more likely to have pre-existing cardiovascular disease (P< 0.001) and respiratory disease (P = 0.003).

Age (P < 0.001) and TBSA (P< 0.001) were the only independent variables that demonstrated a statistically significant higher likelihood of death. Being underweight conferred an odds ratio of 2.569 with respect to mortality but it did not rise to statistical significance. Obesity was not an independent risk factor for increased mortality. Underweight patients were more likely be discharged to a rehabilitation or skilled nursing facility and less likely to be sent home (P=0.002). There was no difference in TBSA, total length of stay, ICU or ventilator days amongst the six groups.

**Conclusions:**

Having a low or high BMI does not increase the mortality rate of hospitalized burn patients. However, being underweight is a risk factor for a hospital disposition other than home.

**Applicability of Research to Practice:**

Abnormal BMI alone does not confer an increased mortality risk in burn patients. More studies need to be performed to investigate other adverse events related to BMI.

